# Prevalence of anemia and sociodemographic characteristics among pregnant and non-pregnant women in southwest China: a longitudinal observational study

**DOI:** 10.1186/s12884-020-03222-1

**Published:** 2020-09-14

**Authors:** Yu Wu, Hanfeng Ye, Jihong Liu, Qiuyue Ma, Yanling Yuan, Qian Pang, Jue Liu, Cai Kong, Min Liu

**Affiliations:** 1 Department of Epidemiology and Biostatics, School of Public Health, Peking University, No.38, Xueyuan Road, Haidian District, 100191 Beijing, China; 2 Yunnan Population and Family Planning Research Institute, No.146, Qingnian Road, Wuhua District, Yunnan 650021 Kunming, China; 3 The Second Affiliated Hospital of Kunming Medical University, No.1168, Chunrong west Road, Chenggong District, Yunnan 650500 Kunming, China

**Keywords:** Anemia, Women`s health, Prevalence, Southwest China

## Abstract

**Background:**

Globally, the prevalence of anemia among women of reproductive age is about 29.4%, and anemia impacts about 40% of pregnant women and more than 20% of non-pregnant women. We conducted a longitudinal observational study of anemia in pregnant and non-pregnant women, and analyzed the association between the prevalence of anemia and sociodemographic characteristics of women in southwest China.

**Methods:**

This study was a longitudinal observational study which involved 640,672 women aged 18–49 years from 129 counties in southwest China. Data were from databases of National Free Preconception Health Examination Project (NFPHEP) and electronic medical records of local hospitals. We adjusted the diagnostic thresholds of anemia for altitude. The prevalence of anemia was expressed in percentages and 95% confidence intervals (95% CI). The association between the prevalence of anemia and sociodemographic characteristics of pregnant and non-pregnant women were analyzed using univariate and multivariate logistic regression method, expressed in crude odds ratio (cOR), adjusted odds ratio (aOR) and 95%CI.

**Results:**

Of the 640,672 participants, 121,254 women suffered from anemia, with the prevalence of 18.9% (95%CI: 18.8–19.0%). From 2014 to 2018, the prevalence of anemia declines from 23.0–16.4%.The prevalence was 21.6% in the first trimester, higher than women in non-pregnancy (17.4%) and women in the third trimester (10.5%). Results from the multivariable logistic regression showed that women aged 18–20 (aOR = 1.28) or over 35 years old (aOR = 1.07), being farmers (aOR = 1.42), being ethnic minorities (aOR: 1.19 ~ 1.73), during the first trimester (aOR = 1.32) were more likely to be anemic.

**Conclusions:**

Although the anemia prevalence of women of reproductive age has been decreasing in recent years, the prevalence of anemia is still high in pregnant and non-pregnant women in southwest China, especially during the first trimester. Women who were older or younger, being farmers, being ethnic minorities were at high risk of anemia. Anemia in women of reproductive age cannot be neglected.

## Background

Anemia is a global public health problem. Women of reproductive age are particularly at risk [1, 2]. Anemia can be caused by both nutritional and non-nutritional factors, with iron deficiency being the most common cause [3]. Previous studies reported that anemia affected about 40% of pregnant women and more than 20% of non-pregnant women [1]. Due to the increased demand for iron, normal diet cannot meet the demand of some pregnant women for iron, especially women with an already established iron deficiency [4]. Anemia not only can increase the risk of adverse pregnancy outcomes [5–7], but may reduce cognitive function [8], and reduce work efficiency [9].

In 2011, World Health Organization (WHO) reported that the global prevalence of anemia for all women of reproductive age was 29.4%, with 38.2% in pregnant women and 29.0% in non-pregnant women [10]. The anemia prevalence was about 20% in Chinese women of reproductive age, with 22% in pregnant women, and 19% in non-pregnant women [10]. The 2010–2012 Chinese Nutrition and Health Surveillance showed that the prevalence of anemia among pregnant women and non-pregnant women in China was 17.2% [11] and 15.4% [12], respectively. Our previous large-sample population-based study showed that in 2012, the anemia prevalence was 24.8% among non-pregnant women of reproductive age in rural areas of China [13], and the severe anemia prevalence was 0.24% [14]. A meta-analysis showed that the gestational anemia prevalence in Chinese women from 2012 to 2016 was 19.9% (95% confidence intervals (CI): 16.3%-23.5%) [15]. Although the anemia prevalence in women of reproductive age in China is lower than the global average, the anemia among women of reproductive age cannot be neglected.

China is a large country with a population of nearly 350 million women of reproductive age. It is of great public health significance to improve the anemia among women of reproductive age. Most of the previous studies focused on the prevalence of anemia among pregnant women [11, 15, 16], and a few studies focused on the prevalence of anemia among non-pregnant women [13, 17], but there was a lack of large-sample studies about anemia covering both pregnant and non-pregnant women. We did a large-longitudinal observational study covering 129 counties in southwest China which is a multi-ethnic region with one-seventh of China’s population, using the data from National Free Preconception Health Examination Project (NFPHEP) and electronic medical records of local hospitals to analyze the prevalence of anemia and sociodemographic characteristics of pregnant and non-pregnant women in this region, so as to provide evidence for improving the anemia status of women of reproductive age.

## Methods

### Study design and participants

We conducted a longitudinal observational study, using data from NFPHEP and electronic medical records of local hospitals. Inclusion criteria for study entrance were women aged 18–49 who intended to become pregnant within the next six months or had been already pregnant. Exclusion criteria were women without any hemoglobin records. 640,672 women aged 18–49 years from 129 counties in southwest China from 2014 to 2018 were involved. All the participants were given written informed consent forms before enrollment. A standardized questionnaire was used by local maternal and child health care personnel and obstetricians to collect information, and venous blood was extracted for relevant laboratory tests. The information included sociodemographic characteristics (such as age, occupation and ethnic), history of childbirth, chronic diseases (such as hypertension, diabetes and thyroid diseases) of the participants was collected from questionnaire. Hemoglobin detection was performed by qualified and trained professionals in the laboratory of a qualified medical institution, and the concentration of hemoglobin was measured using the cyanide methemoglobin method by trained medical staff in all the surveys and all years. Only a single Hb data was available for each participant, and there was no repeat testing.

In this study, according to the age distributions, all the participants were divided into five groups as follows: 18–20 years, 21–25 years, 26–30 years, 31–35 years, and over 35 years old. Again, according to the occupation distributions, the participants were divided into three groups: workers, farmers and others. The ethnic was divided into five groups: Han, Yi, Hani, Miao and others minorities, among which Yi, Hani, Miao and other minorities were ethnic minorities. Pregnancy stages were defined based on the results of the urine pregnancy test at the time the participants entered the program. Women who had a negative urine pregnancy test were defined as non-pregnant, and women who had a positive urine pregnancy test were defined as pregnant. The whole gestation process was calculated from the first day of the last menstruation. The gestational week before the 13th weekend was the first trimester, gestational week at the 28th week and later was the third trimester [18].

In this study, anemia was defined as lower than 110 g/L for pregnant women and lower than 120 g/L for non-pregnant women, recommended by the WHO [19]. We also adjusted the thresholds for altitude since it was related to hemoglobin (Hb) concentration. Adjustments of anemia thresholds for people living at altitudes higher than 1000 m were recommended by the WHO [19]: thresholds plus 2 g/L for people living at altitudes between 1000 m and 1500 m; plus 5 g/L for those living at altitudes between 1500 m and 2000 m; plus 8 g/L at 2000–2500 m; plus 13 g/L at 2500–3000 m; plus 19 g/L at 3000–3500 m; plus 27 g/L at 3500–4000 m; 35 g/L at 4000–4500 m; and plus 45 g/L at altitudes higher than 4500 m.

### Statistical analysis

First, the descriptive analysis was performed for all the data. Sociodemographic characteristics (including age, occupation, ethnic) and pregnancy stage were described by proportion. The prevalence of anemia was reported as percentages and 95%CI. Second, after controlling the investigation year and the history of childbirth, logistic regression was used to analyze the association between sociodemographic characteristics and the prevalence of anemia among women in different pregnancy stage. Third, univariate and multivariable logistic regressions were used to examine the association of sociodemographic characteristics with the prevalence of anemia among all participants.

### Sensitivity analysis

Considering that chronic diseases may have a certain impact on women’s metabolism, three other variables were included in the sensitivity analysis: hypertension, diabetes and thyroid diseases. In the subgroup analysis, participants were stratified by history of childbirth to detect the stability of associations, because women with different childbirth history may have different distributions of Hb. The significant difference was *P* < 0.05. All statistical analysis was performed on the SPSS21.0 software.

## Results

### Sociodemographic characteristics and prevalence of anemia in women of reproductive age

A total of 640,672 participants were included in this study, with 351,980 (54.9%) non-pregnant women, 268,014 (41.8%) women in the first trimester, and 20,678 (3.2%) women in the third trimester. Sociodemographic characteristics of the participants were as follows: 434,140(67.7%) were aged 21–30 years; 556,838(86.9%) were farmers; 413,746(64.6%) were the Han, 226,926(35.4%) were ethnic minorities (Table 1).

**Table 1:** Socio-demographic characteristics anemia prevalence among women of reproductive age in 2014–2018, southwest China

	Number	%	Anemia(n)	Prevalence of Anemia(%(95%CI))
**All participants**	640,672	100.00	121,254	18.9(18.8,19.0)
**Stage**				
Non-pregnancy	351,980	54.9	61,193	17.4(17.3,17.5)
First trimester	268,014	41.8	57,883	21.6(21.4,21.8)
Third trimester	20,678	3.2	2178	10.5(10.1,11.0)
**Age group, years**				
18–20	54,792	8.6	13,007	23.7(23.4,24.1)
21–25	223,074	34.8	42,720	19.2(19.0,19.3)
26–30	211,066	32.9	37,577	17.8(17.6,18.0)
31–35	90,235	14.1	16,681	18.5(18.2,18.8)
> 35	61,505	9.6	11,259	18.3(18.0,18.6)
**Occupation**				
Worker	51,277	8.0	7164	14.0(13.7,14.3)
Farmer	556,838	86.9	109,567	19.7(19.6,19.8)
Others	32,557	5.1	4523	13.9(13.5,14.3)
**Ethnic**				
Han	413,746	64.6	70,297	17.0(16.9,17.1)
Yi	74,464	11.6	15,204	20.4(20.1,20.7)
Hani	21,277	3.3	5654	26.6(26.0,27.2)
Miao	18,214	2.8	4219	23.2(22.6,23.8)
Others	112,971	17.6	25,880	22.9(22.7,23.2)

**Fig. 1:**
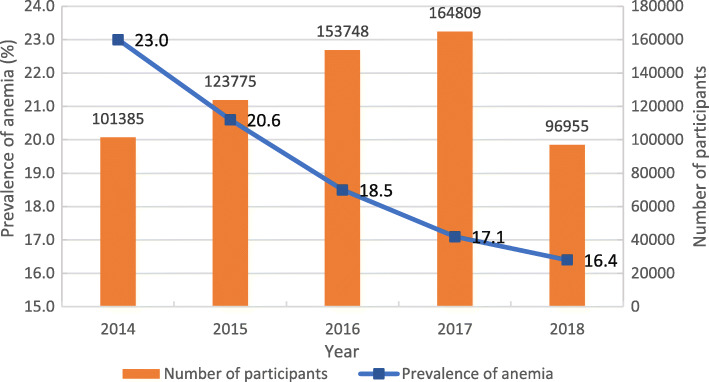
Number of participants and the prevalence of anemia among women of reproductive age

Of the 640,672 participants, 121,254 suffered anemia, with an overall prevalence of 18.9% (95%CI: 18.8%-19.0%). From 2014 to 2018, the prevalence of anemia among women of reproductive age in southwest China decreased from 23.0% in 2014 to 16.4% in 2018 (Fig. 1).

Among the 640,672 participants, the prevalence of anemia was 21.6% (95%CI: 21.4%-21.8%) among women in the first trimester, higher than non-pregnant women (17.4%, 95%CI: 17.3%-17.5%) and women in the third trimester (10.5%, 95%CI: 10.1%-11.0%). The prevalence was significantly higher in those 18–20 years old (23.7%) than in other age groups (17.8–19.2%). The prevalence in farmers (19.7%) was significantly higher than in workers (14.0%). The prevalence of anemia in Yi (20.4%), Hani (26.6%), Miao (23.2%) and other minorities (22.9%) women was significantly higher than that in Han nationality (17.0%, 95%CI: 16.9%-17.1%, Table 1).

### Association between sociodemographic characteristics and the prevalence of anemia in women of different pregnancy stage

**Fig. 2:**
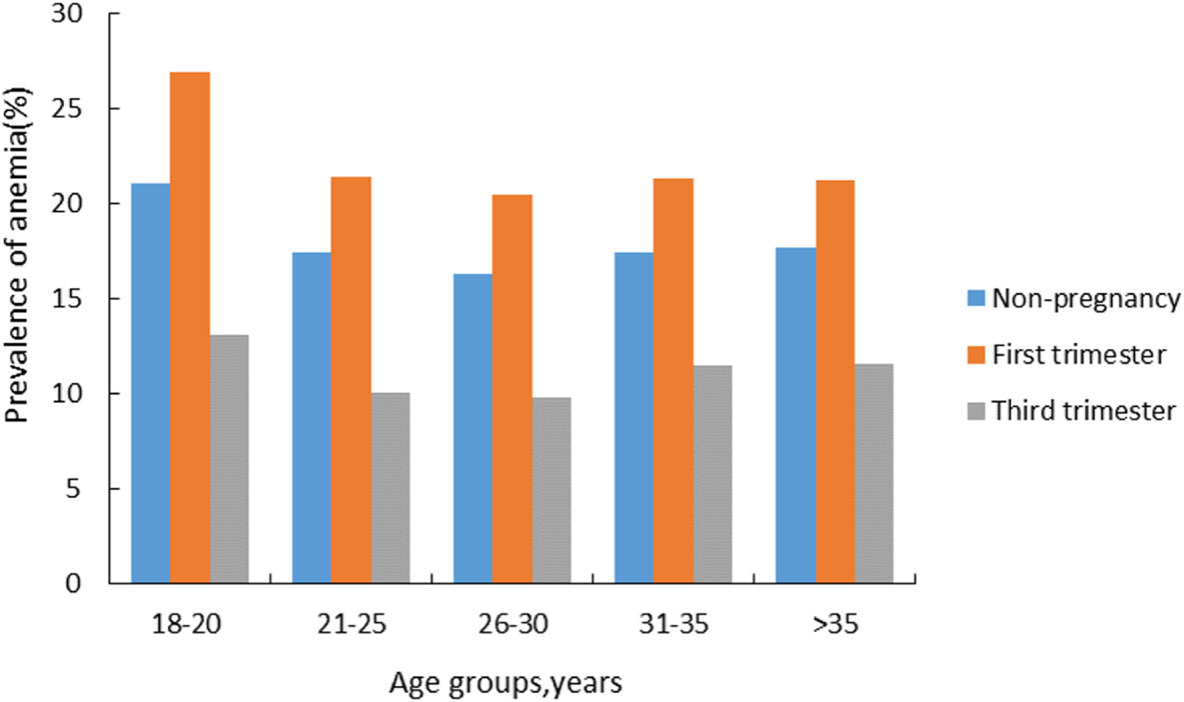
Age-specific prevalence of anemia in different stages of pregnancy in 2014-2018, southwest China

We stratified women according to whether they were in non-pregnancy, the first trimester and the third trimester. The results showed that among women of reproductive age with different characteristics, the prevalence of anemia in women during the first trimester was higher than that in non-pregnant women, and the prevalence in women during the third trimester was lower than that in non-pregnant women. The differences were significant in 18–20, 21–25, 26–30, 31–35 and over 35 years old age groups (Fig. 2).

Regardless of whether women were pregnant or not, women aged 18–20 years, being farmers, being ethnic minorities were under higher risk for anemia (*P* < 0.05, Table 2). Compared with women aged 26–30 years old, women aged 18–20 years were more likely to develop anemia. Compared with workers, farmers were more likely to suffer anemia. The prevalence of ethnic minorities were all higher than that of Han nationality (Table 2).

**Table 2:** Association between the prevalence of anemia and socio-demographic characteristics among women in non-pregnancy, the first trimester and the third trimester

	Non-pregnancy			First trimester			Third trimester		
	**Number**	**Anemia,n(%)**	**aOR**^**a**^**(95%CI)**	**Number**	**Anemia,n(%)**	**aOR**^**a**^**(95%CI)**	**Number**	**Anemia,n(%)**	**aOR**^**a**^**(95%CI)**
**All participants**	351,980	61,193(17.4)	-	268,014	57,883(21.6)	-	20,678	2178(10.5)	-
**Age group, years**									
18–20	28,947	6113(21.1)	1.28(1.24,1.32)	25,464	6844(26.9)	1.33(1.29,1.37)	381	50(13.1)	1.33(1.29,1.37)
21–25	117,023	20,395(17.4)	1.03(1.01,1.05)	102,668	21,982(21.4)	1.00(0.98,1.03)	3383	343(10.1)	1.00(0.98,1.03)
26–30	110,488	17,968(16.3)	1.00	91,018	18,672(20.5)	1.00	9560	937(9.8)	1.00
31–35	51,325	8908(17.4)	1.02(0.99 ,1.05)	33,826	7199(21.3)	1.03(0.99,1.06)	5084	584(11.5)	1.03(0.99,1.06)
> 35	44,197	7809(17.7)	1.05(1.03,1.09)	15,038	3186(21.2)	1.01(0.97,1.06)	2270	264(11.6)	1.01(0.97,1.06)
**Occupation**									
Worker	26,243	3420(13.0)	1.00	20,466	3308(16.2)	1.00	4568	436(9.5)	1.00
Farmer	310,898	55,732(17.9)	1.34(1.29,1.40)	236,570	52,695(22.3)	1.38(1.33,1.44)	9370	1140(12.2)	1.38(1.33,1.44)
Others	14,839	2041(13.8)	1.02(0.96,1.08)	10,978	1880(17.1)	1.03(0.97,1.10)	6740	602(8.9)	1.03(0.97,1.10)
**Ethnic**									
Han	225,698	35,190(15.6)	1.00	168,523	33,057(19.6)	1.00	19,525	2050(10.5)	1.00
Others	126,282	26,003(20.6)	1.38(1.35,1.40)	99,491	24,826(25.0)	1.33(1.30,1.35)	1153	128(11.1)	1.33(1.30,1.35)

^a^Multivariate model adjusted for age, occupation, ethnic, investigation year and history of childbirth among women of reproductive age

The age was taken as the reference by the age group of 26–30 years, the occupation was taken as the reference by workers, the ethnic was taken as the reference by the Han

For non-pregnant women, without a history of pregnancy or childbirth means that the woman has never been pregnant or given birth; with a history of pregnancy or childbirth means that the woman has been pregnant or given birth. For pregnant women, without a history of pregnancy or childbirth means that the woman has never been pregnant or given birth before the current pregnancy; with a history of pregnancy or childbirth means that the woman has been pregnant or given birth before the current pregnancy

### Association between sociodemographic characteristics and the prevalence of anemia in pregnant and non-pregnant women

After controlling the history of childbirth and investigation year, women in the first trimester(aOR = 1.32), aged 18–20 years old(aOR = 1.28) and over 35 years old(aOR = 1.07), working as farmers(aOR = 1.42), of minorities(aOR: 1.19–1.73) were related to a higher anemia prevalence (Table 3). Women in the first trimester (aOR = 1.32, 95%CI: 1.31–1.34) were more likely to have anemia than non-pregnant women, but women in the third trimester were less likely to have anemia (aOR = 0.82, 95%CI: 0.78–0.87). Compared with women aged 26–30 years, women in other age groups were more likely to develop anemia (aOR: 1.01–1.28). Compared with workers, farmers were more likely to be anemic (aOR = 1.42, 95%CI: 1.36–1.49). The prevalence of anemia among the ethnic minorities (aOR: 1.19–1.73) were all higher than that among the Han.

**Table 3:** Association between the prevalence of anemia and socio-demographic characteristics among women of reproductive age in 2014–2018, southwest China

	Number	Anemia,n(%)	cOR(95%CI)	aOR^a^(95%CI)
**All participants**	640,672	121,254(18.9)	-	-
**Stage**				
Non-pregnancy	351,980	61,193(17.4)	1.00	1.00
First trimester	268,014	57,883(21.6)	1.31(1.29,1.33)	1.32(1.31,1.34)
Third trimester	20,678	2178(10.5)	0.56(0.54,0.59)	0.82(0.78,0.87)
**Age group, years**				
18–20	54,792	13,007(23.7)	1.38(1.35,1.42)	1.28(1.25,1.31)
21–25	223,074	42,720(19.2)	1.02(1.01,1.04)	1.01(1.00,1.02)
26–30	211,066	37,577(17.8)	1.00	1.00
31–35	90,235	16,681(18.5)	1.01(0.98,1.03)	1.05(1.03,1.07)
> 35	61,505	11,259(18.3)	1.00(0.97,1.02)	1.07(1.05,1.10)
**Occupation**				
Worker	51,277	7164(14.0)	1.00	1.00
Farmer	556,838	109,567(19.7)	1.51(1.47,1.55)	1.42(1.36,1.49)
Others	32,557	4523(13.9)	0.99(0.95,1.03)	1.04(0.99,1.09)
**Ethnic**				
Han	413,746	70,297(17.0)	1.00	1.00
Yi	74,464	15,204(20.4)	1.25(1.23,1.28)	1.19(1.17,1.21)
Hani	21,277	5654(26.6)	1.77(1.71,1.83)	1.73(1.67,1.78)
Miao	18,214	4219(23.2)	1.47(1.42,1.53)	1.33(1.29,1.38)
Others	112,971	25,880(22.9)	1.45(1.43,1.48)	1.41(1.39,1.43)

^a^Multivariate model adjusted for pregnancy stage, age, occupation, ethnic, investigation year and history of childbirth among women of reproductive age

The stage was taken as the reference by non-pregnancy, the age was taken as the reference by the age group of 26–30 years, the occupation was taken as the reference by workers, the ethnic was taken as the reference by the Han

### Sensitivity analysis

Considering that chronic diseases may have a certain impact on women’s metabolism, three other variables were included in the sensitivity analysis: hypertension, diabetes and thyroid diseases. The results revealed that women in the first trimester (aOR = 1.33), aged 18–20 years old (aOR = 1.31), and over 35 years old (aOR = 1.05), working as farmers(aOR = 1.36), of minorities(aOR: 1.18–1.72) were related to a higher anemia prevalence. (Table 4).

**Table 4:** Association between the prevalence of anemia and sociodemographic characteristics among women of reproductive age after controlling the chronic diseases

	aOR^a^	95%CI^a^				P
**All participants**						
** Stage**						
Non-pregnancy	1.00	-		-		-
First trimester	1.33	1.32		1.35		< 0.001
Third trimester	0.76	0.72		0.80		< 0.001
** Age group, years**						
18–20	1.31	1.28		1.34		< 0.001
21–25	1.02	1.00		1.03		0.026
26–30	1.00	-		-		-
31–35	1.03	1.00		1.05		0.015
> 35	1.05	1.02		1.07		< 0.001
** Occupation**						
Worker	1.00	-		-		-
Farmer	1.36	1.33		1.40		< 0.001
Others	1.01	0.97		1.05		0.544
** Ethnic**						
Han	1.00	-		-		-
Yi	1.18	1.16		1.21		< 0.001
Hani	1.72	1.66		1.77		< 0.001
Miao	1.32	1.27		1.37		< 0.001
Others	1.40	1.38		1.43		< 0.001

^a^Multivariate model adjusted for pregnancy stage, age, occupation, ethnic, investigation year, history of childbirth and chronic diseases among women of reproductive age

The stage was taken as the reference by non-pregnancy, the age was taken as the reference by the age group of 26–30 years, the occupation was taken as the reference by workers, the ethnic was taken as the reference by the Han

Analysis stratified by history of childbirth was also performed. The results revealed that regardless of the history of childbirth, the anemia prevalence in women during the first trimester was higher than that in non-pregnant women, and the anemia prevalence in women during the third trimester was lower than that in non-pregnant women. Women aged 18–20 years and over 35 years had higher risks of suffering anemia than women aged 25–30 years. The prevalence of anemia in farmers was higher than that in workers. Women of ethnic minorities were more likely to suffer anemia than the Han (*P* < 0.05, Table 5).

**Table 5:** Prevalence of anemia in different characteristics among women of reproductive age, stratified by the history of childbirth in 2014–2018, southwest China

	Without history of childbirth				With history of childbirth			
	**Number**	**Anemia,n(%)**	**cOR(95%CI)**	**aOR**^**a**^**(95%CI)**	**Number**	**Anemia,n(%)**	**cOR(95%CI)**	**aOR**^**a**^**(95%CI)**
**All participants**	253,906	46,062(18.1)	-	-	386,766	75,192(19.4)	-	-
**Stage**								
Non-pregnancy	113,575	17,957(15.8)	1.00	1.00	238,405	43,236(18.1)	1.00	1.00
First trimester	126,543	26,705(21.1)	1.42(1.40,1.45)	1.41(1.38,1.44)	141,471	31,178(22.0)	1.28(1.26,1.30)	1.27(1.25,1.29)
Third trimester	13,788	1400(10.2)	0.60(0.57,0.64)	0.77(0.72,0.81)	6890	778(11.3)	0.58(0.53,0.62)	0.69(0.64,0.75)
**Age group, years**								
18–20	32,301	7907(24.5)	1.60(1.55,1.65)	1.37(1.33,1.41)	22,491	5100(22.7)	1.23(1.18,1.27)	1.21(1.17,1.25)
21–25	119,045	21,490(18.1)	1.04(1.01,1.06)	0.98(0.96,1.01)	104,029	21,230(20.4)	1.06(1.03,1.08)	1.05(1.03,1.07)
26–30	79,903	12,537(15.7)	1.00	1.00	131,163	25,040(19.1)	1.00	1.00
31–35	16,388	2921(17.8)	1.07(1.02,1.13)	1.18(1.12,1.24)	73,847	13,770(18.6)	0.95(0.93,0.98)	0.99(0.96,1.01)
> 35	6269	1207(19.3)	1.17(1.10,1.25)	1.25(1.17,1.33)	55,236	10,052(18.2)	0.93(0.91,0.95)	1.00(0.98,1.03)
**Occupation**								
Worker	30,935	3767(12.2)	1.00	1.00	20,342	3397(16.7)	1.00	1.00
Farmer	204,768	40,130(19.6)	1.76(1.70,1.82)	1.76(1.70,1.82)	352,070	69,437(19.7)	1.23(1.18,1.27)	1.13(1.09,1.17)
Others	18,203	2165(11.9)	0.97(0.92,1.03)	0.97(0.92,1.03)	14,354	2358(16.4)	0.98(0.93,1.04)	0.99(0.93,1.05)
**Ethnic**								
Han	171,560	28,353(16.5)	1.00	1.00	242,186	41,944(17.3)	1.00	1.00
Others	253,906	17,709(21.5)	1.38(1.36,1.41)	1.28(1.26,1.31)	144,580	33,248(23.0)	1.43(1.40,1.45)	1.39(1.37,1.41)

^a^Multivariate model adjusted for pregnancy stage, age, occupation, ethnic and investigation year among women of reproductive age

The stage was taken as the reference by non-pregnancy, the age was taken as the reference by the age group of 26–30 years, the occupation was taken as the reference by workers, and the ethnic was taken as the reference by the Han

## Discussion

Anemia in women of reproductive age is a global public health concern [2]. In 2011, WHO reported that the global prevalence of anemia for all women of reproductive age was 29.4% (95%CI: 24.5–35.0%), with the highest prevalence in Africa (37.6%), the lowest in North America (12.4%), and about 31.9% in Asia where China was located [10]. In our study, 640,672 women of reproductive age in southwest China were involved, and the overall prevalence of anemia appeared as 18.9% (95%CI: 18.8–19.0%). Among them, the prevalence of anemia in non-pregnant women was 17.4%, in women during the first trimester was 21.6%, and in women during the third trimester was 10.5%, similar to the results of national studies (Pregnancy: 17.2%-42.1% [11, 15]), Non-pregnancy: 15.4%-35.6% [12, 13]), also similar to the results in Guangdong coastal areas (20.1% [20]), Hebei province (11%-16% [21]) and other regional studies. Our study also found that the prevalence of anemia in women of reproductive age had significant declined from 23.0% in 2014 to 16.4% in 2018. In recent years, the anemia status of women in China had been improved obviously, which might be related to the economic development, the improvement of people’s living standards and the improvement of the nutritional status of women of reproductive age in China.

Anemia is more prevalent in women of reproductive age [19]. A Mexican study showed that the national prevalence of anemia in pregnant women was 17.9% (95%CI: 13.5%-23.3%) higher than that in non-pregnant women (11.6%, 95%CI: 10.9%-12.4%) [22]. Lin et al. [16] conducted a multicenter retrospective study in developed areas of China in 2018, showing that among 43,403 pregnant women, the prevalence of anemia was higher in the 2nd trimester (14.7%) and 3rd trimester (16.6%) than in the 1st trimester (2.7%). In our study, the prevalence of anemia in first trimester was 21.6%, significantly higher than that in non-pregnancy (17.4%, *P* < 0.05). The results were higher than those of the Mexican study and higher than the anemia prevalence among pregnant women in developed areas of China. Our study also found that the anemia prevalence in the third trimester was lower than that in non-pregnant women. This may be related to the high coverage of prenatal care in China (97.8% [23]). In China, prenatal care has been incorporated into the national basic public health service, and pregnant women can receive at least five free prenatal examinations provided by the government during pregnancy. Once a pregnant woman is found to have anemia during prenatal examinations, she will be treated promptly, and then reduce the prevalence of anemia.

Previous studies noticed that demographic characteristics, such as age was associated with the prevalence of anemia in women [17, 24–26]. Gupta et al. [17] found that non-pregnant women 35–49 years of age had a significantly higher prevalence of anemia than non-pregnant women 20–34 years of age. Shamah-levy et al. [18] also showed that in the two National Nutrition and Health Surveys in 1999 and 2006, the anemia in women aged 36–49 years was higher than that in women aged 35 years and below. Five National Health and Nutrition Examination Surveys circles between 2003 and 2012 in the United States showed that the anemia prevalence among women of reproductive age peaked at 40–49 age groups (6.5%, 95%CI: 5.5%-7.5%) [25]. A European study showed that, being younger than 20 increased the chances of anemia (aOR = 1.4, 95%CI: 1.1–1.9) [26]. In our study, the prevalence of anemia were significantly higher in women aged 18–20 years and over 35 years than in women aged 25–30 (under 20 years: aOR = 1.28; over 35 years: aOR = 1.07). When women of reproductive age are too young, risk factors include incomplete development of organs and tissues, unmarried status, low level of education, poor financial conditions, and low body weight, which can lead to notable increases in the incidence of anemia [27]. Furthermore, the increased risk of anemia among younger pregnant women could be due to unplanned pregnancy and suboptimal nutritional status prior to conception [28], leading to a higher prevalence of anemia than other age groups. For older women, the etiology of anemia is complex and ranges from bone marrow failure syndromes to chronic kidney disease, and from nutritional deficiencies to inflammatory processes including inflammaging in immunosenescence [29]. After the implementation of the Two-child policy in China, the proportion of older women has increased, which indicates that anemia among older women should be paid more attention by women themselves and relevant institutions.

Previous studies have shown that there were racial differences in hemoglobin distribution [12], and race was associated with the prevalence of anemia [17]. A study in the United States showed that significant differences in the prevalence of iron deficiency anemia existed by race/ethnicity. Non-Hispanic blacks had a higher prevalence of iron deficiency anemia than non-Hispanic whites, Mexican-Americans and other races, which was due to the differences in socio-economic status [17]. China has 56 ethnic groups, with Chinese Han the majority. There are substantial differences in genetic background, culture, socioeconomic levels, climate and geographic features of the residential area, lifestyle, and dietary pattern among certain ethnic groups [30]. Xu et al. [31] found that the anemia prevalence among minority pregnant women in southern rural area of Guizhou province was 23.96%, higher than the overall anemia prevalence of Chinese pregnant women (17.20%).Xu et al. [32] found that among women of reproductive age in six counties of Jiangsu and Zhejiang province in China, the prevalence of anemia in minority women was higher than that in Han nationality (51.3% vs. 42.8%). Our study was conducted in southwest China, which is a multi-ethnic region, and found that the anemia prevalence in minority women was higher than that in Han nationality. Most ethnic minority women live in remote areas with lower education level, unique dietary habits and genetic background [30]. In addition, some women of reproductive age lack the knowledge of prenatal health care and are not willing to improve their nutritional status and seek health care services [33]. For some ethnic minorities, where the prevalence of inherited haemoglobinopathies was high, the prevalence of anemia might be increased. Zhang et al. reported that the Dai (25.94%), Zhuang (17.23%), and Hani (5.16%) had a higher morbidity prevalence of alleles of thalassemia than the Han (2.56%) [34]. Yi people mainly eat a plant-based diet, and their high intake of phytic acid may lead to low iron absorption [35]. Most of the Miao people live close to the mountains, and the transportation is very inconvenient. The diet is mainly corn, and the intake of meat, fruits and vegetables is insufficient, which leads to the iron absorption barrier and causes the increase of the anemia prevalence [36]. In addition, the Miao population has the highest infection prevalence of soil-borne nematodes [37], which may also be the reason for the high anemia prevalence. In our study, the prevalence of anemia in farmers was 1.42 times higher than that in workers. The study of Ma et al. [13] on non–pregnant women of reproductive age in rural areas of China also showed that the prevalence of anemia in farmers was significantly higher than that in workers and other occupations (*P* < 0.05). This might be related to the inadequate access to health services, and farmers were exposed to higher risk of disease [12]. Meanwhile, farmers are usually of low socioeconomic status, which represented insufficient diet, limited education, and they were exposed to occupational hazards, including extreme weather conditions, dangerous chemicals, schistosomiasis, and hookworm infections [12, 38, 39], which may lead them to suffer from anemia.

The physiological haemodilution in pregnancy results in alterations of hematological parameters particularly in a reduction of hemoglobin concentration [4]. Previous studies showed that parity negatively associated with hemoglobin concentration [40, 41]. Compared to the non-pregnant state, every pregnancy carries an increased risk of hemorrhage before, during, and after delivery, especially during short pregnancy intervals, because of limited recovery time. Therefore, higher parity exposes women more frequently to periods of hemorrhage risk [40]. A study in Mexico showed that women with a history of childbirth had a higher prevalence of anemia than women without a history of childbirth (OR: 1.5–1.8) [42]. An Ethiopian study also showed that, mothers with five and above history of parity were found with increased odds of anemia compared to those who had parity of less than two (OR = 4.20, 95%CI: 1.14–15.52) [43]. Our study also found that women with a history of childbirth were more likely to suffer anemia than women without a history of childbirth, which was consistent with the above findings.

The strength of our research was that, to our knowledge, this was the largest population-based longitudinal observational study of anemia in China which covered both pregnant and non-pregnant women. Prior to our study, most of the published literatures in China focused on anemia during pregnancy, and there was no study about anemia in both pregnant and non-pregnant women of reproductive age. We used the data from NFPHEP and electronic medical records of local hospitals from 129 counties in southwest China to obtain a better representation of the anemia prevalence in women of reproductive age in southwest China. At the same time, we adjusted the thresholds for altitude recommended by WHO, enabling our results to compare with other study of the same kind.

Limitations in our study included the following: the sample size of women in the third trimester involved in this study was relatively small, which accounted for a small proportion of the study participants. Therefore, the anemia prevalence in the third trimester in our study might be underestimated. In our study, there were few variables related to reproductive status of women, and only the pregnancy history and childbirth history were included, and for possible factors that affected anemia, such as menstrual quantity, the use of intrauterine device and diet nutrition were not included in the analysis, to a certain extent, may affect the interpretation of the results.

## Conclusions

In recent years, the coverage of prenatal care services in China has expanded constantly. Women pay more attention to nutrition during pregnancy, so the anemia condition of women is improving. Although the prevalence of anemia has had significant declines in recent years, the prevalence among women in southwest China was still high from 2014 to 2018. This continues to be a public health problem affecting women’s health. Our study found that the prevalence in women during the first trimester was higher than that in non-pregnant women, and a certain percentage of women in the third trimester still had anemia. Women who were older or younger, being farmers, being ethnic minorities were at higher risk of anemia. They should have priority to be paid close attention. Therefore, the nutritional status of women before and during pregnancy should be concerned particularly, which may lead to the reduction of the anemia prevalence in women, the reduction of complications during pregnancy, and the improvement of maternal and child health.

## Data Availability

The data that support the findings of this study are available from Yunnan Population and Family Planning Research Institute and The Second Affiliated Hospital of Kunming Medical University, but restrictions apply to the availability of these data, which were used under license for the current study, and so are not publicly available. Data are however available from the authors upon reasonable request and with permission of Yunnan Population and Family Planning Research Institute and The Second Affiliated Hospital of Kunming Medical University.

## Abbreviations

NFPHEP: National Free Preconception Health Examination Project
WHO: World Health Organization
CI: Confidence intervals
